# Association of Protective *HLA-A* With *HLA-B^∗^27* Positive Ankylosing Spondylitis

**DOI:** 10.3389/fgene.2021.659042

**Published:** 2021-07-15

**Authors:** Jessika Nordin, Mats Pettersson, Lina Hultin Rosenberg, Argyri Mathioudaki, Åsa Karlsson, Eva Murén, Karolina Tandre, Lars Rönnblom, Alf Kastbom, Jan Cedergren, Per Eriksson, Peter Söderkvist, Kerstin Lindblad-Toh, Jennifer R. S. Meadows

**Affiliations:** ^1^Science for Life Laboratory, Department of Medical Biochemistry and Microbiology, Uppsala University, Uppsala, Sweden; ^2^Science for Life Laboratory, Department of Immunology, Genetics and Pathology, Uppsala University, Uppsala, Sweden; ^3^Science for Life Laboratory, Department of Medical Sciences, Uppsala University, Uppsala, Sweden; ^4^Department of Rheumatology, University Hospital Linköping, Linköping, Sweden; ^5^Department of Biomedical and Clinical Sciences, Linköping University, Linköping, Sweden; ^6^Broad Institute of MIT and Harvard, Cambridge, MA, United States

**Keywords:** ankylosing spondylitis, *HLA-B^∗^27* positive, *HLA-A^∗^24:02*, sex biased, major histocompatibility complex, HLA allele typing

## Abstract

**Objectives:**

To further elucidate the role of the MHC in ankylosing spondylitis by typing 17 genes, searching for *HLA-B^∗^27* independent associations and assessing the impact of sex on this male biased disease.

**Methods:**

High-confidence two-field resolution genotyping was performed on 310 cases and 2196 controls using an *n-1* concordance method. Protein-coding variants were called from next-generation sequencing reads using up to four software programs and the consensus result recorded. Logistic regression tests were applied to the dataset as a whole, and also in stratified sets based on sex or *HLA-B^∗^27* status. The amino acids driving association were also examined.

**Results:**

Twenty-five *HLA* protein-coding variants were significantly associated to disease in the population. Three novel protective associations were found in a *HLA-B^∗^27* positive population, *HLA-A^∗^24:02* (OR = 0.4, CI = 0.2–0.7), and HLA-A amino acids Leu95 and Gln156. We identified a key set of seven loci that were common to both sexes, and robust to change in sample size. Stratifying by sex uncovered three novel risk variants restricted to the female population (*HLA-DQA1^∗^04.01, -DQB1^∗^04:02, -DRB1^∗^08:01;* OR = 2.4–3.1). We also uncovered a set of neutral variants in the female population, which in turn conferred strong effects in the male set, highlighting how population composition can lead to the masking of true associations.

**Conclusion:**

Population stratification allowed for a nuanced investigation into the tightly linked MHC region, revealing novel *HLA-B^∗^27* signals as well as replicating previous *HLA-B^∗^27* dependent results. This dissection of signals may help to elucidate sex biased disease predisposition and clinical progression.

## Introduction

Ankylosing spondylitis (AS) is a chronic inflammatory disease defined by the inflammation of the spine and sacroiliac joints, which if left untreated, leads to vertebrae fusion (Cortes et al., 2013). The disease prevalence in Sweden is 0.18% (Exarchou et al., 2015) and Europe in 0.24% (Dean et al., 2014), and contrary to most immunological diseases, AS affects males more often than females [Sweden 1.6:1 (Exarchou et al., 2015), Europe 2–3:1 (Lee et al., 2008)]. Not only is the prevalence of disease different between sexes, but so are the manifestations, e.g., males have greater radiographic changes compared with female patients (Lee et al., 2008; Ward et al., 2009).

Approximately 80% of AS cases are *HLA-B^∗^27* positive, while only a small fraction (<5%) of carriers develop disease (Cortes et al., 2013). This clearly indicates that there are other genetic factors involved in disease predisposition. Together with *HLA-B*, more than 45 genes have been suggested to contribute to disease risk, e.g., *ERAP1*, *IL23R*, and *RUNX3* (Burton et al., 2007; Evans et al., 2011; Cortes et al., 2013; Ranganathan et al., 2017), but combined, these explain less than 30% of the genetic heritability (Burton et al., 2007; Evans et al., 2011; Cortes et al., 2013) of this highly heritable disease (*h*^2^ > 90%, Cortes et al., 2013).

The strongest AS association signal comes from the MHC (Reveille, 2014), a region where the genetic contribution is hard to dissect due to high linkage disequilibrium (LD). Previous studies have indicated that variants in addition to *HLA-B^∗^27* are driving the signal, for example, *HLA-A^∗^02:01* has been associated with *HLA-B^∗^27* positive [odds ratio (OR) = 1.2] (Reveille, 2014; Cortes et al., 2015), as well as *HLA-B^∗^27* negative disease (OR = 1.4) (Reveille, 2014; Cortes et al., 2015). Whereas *HLA*-*B^∗^07:02* (OR = 0.8), -*B^∗^40:01* (OR = 1.2), and *-DRB1^∗^01:03* (OR = 1.2) were shown to be significantly associated with AS in a mixed *HLA-B^∗^27* study, i.e., one containing both *HLA-B^∗^27* positive and negative samples (Cortes et al., 2015). These studies highlight the challenges in assessing disease HLA associations, with inconsistencies in replication partly driven by differing sample ancestry, sample sizes, gene loci considered, genotyping methods and levels of phenotypic information (e.g., sex ratio). For example, the small risk conferred by *HLA-A^∗^02:01* is seldom replicated, likely due to the low odds ratio requiring larger sample sizes (Reveille, 2014). In other examples, associations may be a reflection of *HLA-B^∗^27* carrier status and enriched haplotypic pairs within a population. Several variants within the *HLA-B* locus have reported disease association, but only three have been associated in distinct *HLA-B^∗^27* positive (*HLA*-*B^∗^40:01*) (Cortes et al., 2015), or negative (*HLA*-*B^∗^44* and -*B^∗^49*) (Reveille et al., 2019), populations. The same is true for *MICA*, where *MICA^∗^007:01* has been shown to contribute strong susceptibility to both *HLA-B^∗^27* mixed (OR = 60.7) and negative disease (OR = 9.1) (Zhou et al., 2014). A separate study failed to replicate the latter and claimed that the mixed result may be due to linkage to *HLA-B^∗^27* (Cortes et al., 2018). The first study used lab typing at a two-field resolution (Zhou et al., 2014) while the latter imputed *MICA* with SNP2HLA and reported at one-field resolution (Cortes et al., 2018). Although both examined Caucasian populations of European ancestry, these differed in size and composition (sex distribution was not reported in one study), confounding the comparison (Zhou et al., 2014; Cortes et al., 2018).

While 37 separate genes are referenced in the IPD-IMGT/HLA database (release 3.37.0), to date the maximum number of genes studied in any single study was six, *HLA-A, -B, -C, -DRB1, -DQB1*, and *-DPB1* (Reveille et al., 2019). That analysis of a PCR lab-typed Caucasian population (1948 cases/990 controls) performed analyses on their total dataset, followed by an nested test on only those samples which were *HLA-B^∗^27* negative (Reveille et al., 2019). At least one variant from each gene examined was shown to be significantly associated to disease in the *HLA-B^∗^27* mixed population, with almost two thirds conferring protection (28 variants; OR = 0.3–0.8, risk OR = 1.3–21.4) (Reveille et al., 2019). Fewer associations were detected in the *HLA-B^∗^27* negative population; however five genes (11 variants) were shown to be linked to AS, with the protective contribution dropping to around 50% (Reveille et al., 2019). No similar efforts have been performed in a *HLA-B^∗^27* positive population.

Here we aimed to build the largest typed MHC gene set (17 genes; *HLA-A*, *-B*, *-C*, *-DOA*, *-DOB*, *-DPA1*, *-DPB1*, *-DQA1*, *-DQB1*, *-DRA*, *-DRB1*, *-E*, *-F*, *-G*, *MICA MICB*, and *TAP2*) for a single AS population, and with this data address the following; can we identify novel signals of AS association, are these different between the sexes, and are they independent of *HLA-B^∗^27*.

## Materials and Methods

### Sample Data

Samples were drawn from existing targeted (SweAS and Uppsala Bioresource, Eriksson et al., 2016) or whole genome sequencing (SweGen, Ameur et al., 2017) experiments and used to build the case and control populations (Table 1). Cases were from SweAS (*n* = 310, 26.8% female and 73.2% males) and controls from SweAS (*n* = 381, age and region matched to cases from South East Sweden), SweGen (*n* = 1000, obtained from across Sweden), and the Uppsala Bioresource (*n* = 815, from South East Sweden). In total, 2196 controls were collated, 40.2% were males and 59.8% females (ages for cases and controls summarized in Supplementary Figure 1). Cases were diagnosed according to the modified New York criteria (van der Linden et al., 1984) and four comorbidities (psoriasis, uveitis, peripheral joint involvement and gut involvement) were recorded (Supplementary Figure 2). The SweAS population was enrolled under the ethical approval granted from the Regional Committee of Linköping, Dnrs 2010/182-3 and 98110, whereas ethical approvals for SweGen and the Uppsala Bioresource are as per their cited publications.

**TABLE 1:** Summary of each population tested for the association analyses.

**Set^1^**	**Cases/controls^2^**	**Cases/controls with *HLA-B* genotype^3^ (% *HLA-B27* positive)**	**FDR^4^**
ALL	310/2196	150 (96.8)/1926 (13.9)	9.4 × 10^–4^
F	83/1313	70 (92.9)/1137 (13.5)	1.3 × 10^–3^
M	227/883	179 (98.3)/789 (14.3)	8.8 × 10^–4^
ALL.B27	241/267	241 (100.0)/267 (100.0)	1.7 × 10^–3^
F.B27	65/154	65 (100.0)/154 (100.0)	1.7 × 10^–3^
M.B27	176/113	176 (100.0)/113 (100.0)	2.0 × 10^–3^

^1^Set of samples considered; the complete set (ALL), Male (M) and Female (F) and then that set restricted to HLA-B*27 positive samples.

*^2^The maximum number of samples considered at each gene locus.*

^3^The number of samples with a *HLA-B* genotype, followed by the fraction which were HLA-B*27 positive.

*^4^The set specific false discovery rate (FDR) significance threshold determined via permutation.*

### HLA Variant Typing

An *n-1* concordance method (Nordin et al., 2020) was used to ensure high quality genotyping across the MHC. The inputs for this were raw *HLA* genotype calls generated from four separate software programs, with consensus variant calls reported with 2-field resolution. In brief, this meant that for a variant to be called in the final set, the result had to be identical across three out of four programs (Supplementary Figure 3). As noted previously (Nordin et al., 2020), this procedure can account for software biases, such as reference version and algorithm choice. Called chromosome 6 SNPs were the base data for imputation (SNP2HLA, Jia et al., 2013), whereas reads mapped to chromosome 6, plus unmapped reads, were used as inputs for inference tools [HLA-VBSeq (Nariai et al., 2015), HLAscan (Ka et al., 2017), and HLA-HD (Kawaguchi et al., 2017)]. SNP2HLA, HLA-VBSeq and HLAscan were previously used to genotype SweGen at eight *HLA* genes (Nordin et al., 2020), however this was expanded to 17 genes (*HLA-A*, *-B*, *-C*, *-DOA*, *-DOB*, *-DPA1*, *-DPB1*, *-DQA1*, *-DQB1*, *-DRA*, *-DRB1*, *-E*, *-F*, *-G*, *MICA*, *MICB*, and *TAP2)* with the inclusion of HLA-HD. The impact of biases on genotyping this set of genes was assessed with average read depth across each for the three sample populations [10 bp bins in BEDtools (Quinlan and Hall, 2010) v2.26.0], shared variant availability across software references, and concordance rate between the high confidence set and each software. See Supplementary Methods for information on software algorithms and running conditions.

### Association Tests and Statistical Methods

In order to address the question of sex bias, the dataset was partitioned into three sets; ALL, all samples; F, female samples; M, male samples (Table 1). To test for independence to *HLA-B^∗^27*, each analysis was repeated using only those samples carrying at least one copy of any *HLA-B^∗^27* variant (*HLA-B^∗^27* positive): ALL.B27, F.B27, and M.B27 (Table 1).

Genes were excluded from all six analyses if their genotyping rate was below 80% in ALL. Before the association tests were performed, the potential impact of data missingness was investigated with Fisher’s exact test, and sequencing batch effects were assessed with logistic regression association tests on targeted versus whole genome sequenced controls. Disease association employed logistic regression with an additive model. Sex was shown to be significantly associated with disease status in both the ALL and ALL.B27 populations and was included as a covariate in those tests (*z*-score for proportions, *p*-value < 1.0 × 10^–5^). The association between AS and HLA amino acids in the *HLA-B^∗^27* positive population, or MICA’s transmembrane region (TM) for all six sets, were also explored (Fisher’s exact test, without covariates). This last phase of the association study was conducted per gene and did not consider individuals with missing data. Disease association tests were performed with PyHLA (Fan and Song, 2017). Phenotype permutations (*n* = 1000) were used to determine cohort specific significance thresholds (5% false discovery rate, FDR) for gene, amino acid and TM tests (Table 1). A second threshold (*p*-value < 0.05) was used to identify suggestive gene results, this time compared to Bonferroni adjusted *p*-values (number of variants for that gene^∗^unadjusted *p*-value).

Gene level pair-wise LD was measured using the multi-marker statistic, *x^2^’* (Zhao et al., 2005). Only samples with a 100% genotyping rate for the genes of interest were taken forward for phasing as described previously (PHASE v2.1.1, Stephens et al., 2001; Nordin et al., 2020). Variant level pair-wise LD was calculated with phased inputs (*r*^2^). For significant amino acids, Students *t*-test was used to assess phenotype enrichment (age and C-reactive protein, CRP). The potential consequence of significant amino acids on protein structure were explored with SNPeffect 4.0 (De Baets et al., 2012), MHC motif viewer (Rapin et al., 2010), and visualized with Chimera (Pettersen et al., 2004).

## Results

Seventeen genes were genotyped at twofield resolution with a success rate of between 77 and 100% (Supplementary Table 1). This rate was driven by a combination of data input and software biases, where some genes were genotyped at a higher rate with targeted data than WGS (e.g., *TAP2*) and some gene call rates were affected by variant availability and software choice (Supplementary Table 3 and Supplementary Figures 4–6). The majority of cases were *HLA-B^∗^27* positive (cases/controls 96.8/13.9%, Table 1), and as a group, cases were less polymorphic than controls in terms of variant count, but more heterozygous overall (Supplementary Table 1). The presence of *HLA-B^∗^27* was skewed across the sets, with a slightly higher fraction observed in males compared with females (e.g., cases M/F = 98.3/92.9%, Table 1).

### Association Tests Revealed Female Specific Results

**FIGURE 1:**
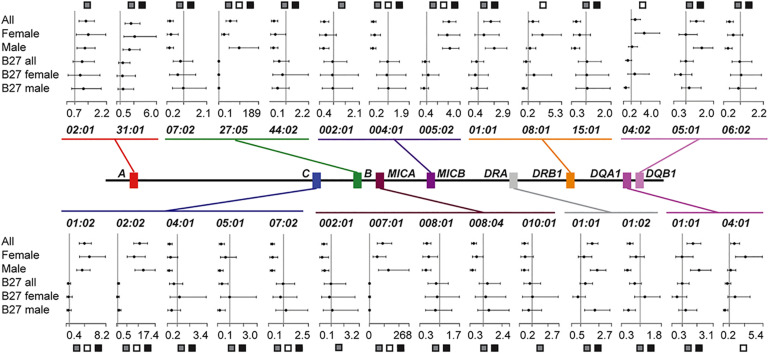
Summary of significant *HLA* protein-coding variant associations and their effect sizes. Plots illustrate the odds ratio (OR) and 95% confidence interval for each of the six datasets considered. An OR of 1 is indicated with a vertical line. The dataset for which the protein-coding variant was significant is indicated with boxes: All (ALL), gray; Female (F), white; Male (M), black.

Of the 15 genes passing quality control (*HLA-DPA1* and *TAP2* excluded), 9 genes (25 protein-coding variants) conferred a significant effect in ALL (Figure 1 and Supplementary Table 3). *HLA-B^∗^27:05* demonstrated the most associated risk (OR = 54.9, *p*-value = 7.1 × 10^–68^), followed by *MICA^∗^007:01* (OR = 89.4, *p*-value = 3.0 × 10^–63^) and -*C^∗^02:02* (OR = 10.6, *p*-value = 6.3 × 10^–61^). Interestingly, *HLA-A* (^∗^*02:01* and ^∗^*31:01*) showed a purely protective profile (Figure 1). Eight variants from *HLA-A, -B, -C, -DQA1, -DQB1* and *–F* were suggestively associated with disease (Supplementary Figure 6).

We next stratified our ALL population by sex to assess if this covariate was masking signals of association. We found that seven protein-coding variants across *HLA-B, -C, MICA*, and *MICB* were significant, irrespective of sex or population size (Figure 1, Datasets ALL, F, and M). Perhaps reflecting ALL dataset composition (73% males), 14 significant variants were common to the ALL and M sets, but none were shared by only the ALL and F populations. For most variants shared between ALL and M (12/14), the OR for F had the same direction of effect, even though it was not significant. However, for some protein-coding variants, e.g., *HLA-DQA1^∗^01:01* and -*DQB1^∗^05:01*, the modest effect conferred in ALL and M (OR = 1.6–1.7) was neutral in F (OR = 1). Intriguingly, three variants were significant only in F, *HLA-DQA1^∗^04:01, -DQB1^∗^04:02* and *-DRB1^∗^08:01* (F *p*-value = 3.4 × 10^–5^–1.3 × 10^–3^, M *p*-value > 0.39, Figure 1 and Supplementary Table 3). Each of these, class II variants conferred additional risk (OR = 2.4–3.1), but with broad confidence intervals (Supplementary Table 3). This result was not only driven by LD with *HLA-B^∗^27* (*r^2^* < 0.01), as the *HLA-DQA1^∗^04:01 -DQB1^∗^04:02 -DRB1^∗^08:01* haplotype was also observed to segregate more frequently with *HLA-B^∗^35:01* in cases than controls (Supplementary Table 4).

The protein-coding variant frequency differences driving these sex specific signals were explored further. For the three F specific significant variants, the delta variant frequency (ΔVF) between cases and controls was greater than 6% (*HLA-DQA1^∗^04:01* = 9.3%, *-DQB1^∗^04:02* = 6.6%, *-DRB1^∗^08:01* = 6.5%). For the same protein-coding variants in M, the ΔVF was < 0.02%, explaining why these signals were not significant in ALL or M. The largest ΔVFs calculated between M and F datasets were for both *DRA* variants (>10%, only considering variants significant in ALL).

### *HLA-B^∗^27* Positive Cohort Reveals Novel *HLA-A* Association

**FIGURE 2:**
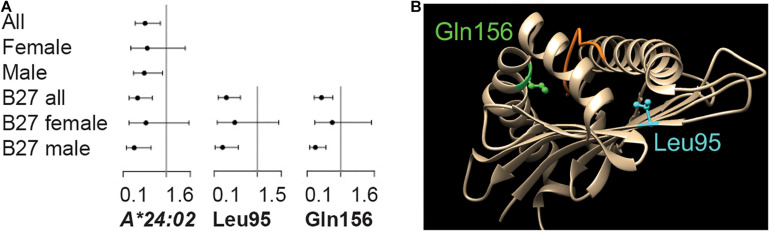
Variants within the *HLA-A* locus are significantly associated with HLA-B*27 positive disease. **(A)** Significant association was observed for protein-coding variant HLA-A*24:02 and amino acid position Leu95 in the ALL cohort and Gln156 in the Male dataset. Odds ratios and 95% confidence intervals are indicated. **(B)** The physical position of each amino acid is indicated.

While the pair-wise LD between the significant MHC genes was low (*x^2^′* < 0.01, Supplementary Table 5), this did not indicate that the individual protein-coding variants across MHC genes were independent. For that question, the *HLA-B^∗^27* positive datasets were tested. The result was a single significant variant from ALL.B27, *HLA-A^∗^24:02* (OR = 0.4, *p*-value = 1.7 × 10^–3^, Figure 2A and Supplementary Table 3). Phasing revealed the *HLA-A^∗^24:02-HLA-B^∗^27:05* combination to be one of the most common haplotypes in either cases or controls for this dataset (haplotype case/control = 3.1/8.1%; Supplementary Table 6). However, *HLA-A^∗^24:02* was observed to be segregating with nine additional *HLA-B* protein-coding variants, none of which were *HLA-B^∗^27* variants (Supplementary Table 7) and the LD between the *HLA-B^∗^27* and *–A^∗^24:02* was negligible (*r^2^* < 0.01). Within the divided sex sets, *HLA-A^∗^24:02* showed suggestive significance within M.B27, along with the risk variant *HLA-DRA^∗^01:01* and protective variant *HLA*-*DRA^∗^01:02* (*p*-value < 3.0 × 10^–3^, Supplementary Table 3). No significant or suggestive associated variants were detected in F.B27.

### Amino Acids in HLA-A Are Significantly Associated With Disease

While no significant associations were observed between disease and MICA TM repeats, tests of association using amino acids across the *HLA-B^∗^27* positive population did resolve divided or underpowered signals from the gene tests. The result was two protective HLA-A amino acids, Ile/Val95Leu (ALL.B27 *p*-value = 3.2 × 10^–4^) and Arg/Leu/Trp156Gln (M.B27 *p*-value = 1.8 × 10^–4^; F.B27 *p*-value > 0.05) (Figure 2A and Supplementary Table 3). For Leu95, the overall ΔVF between cases and controls (13.7%) was reflected in both M.27 and F.27 (17.4% and 8.8% respectively), however, the skew was more pronounced for Gln156 (ALL.B27 = 18.0%, M.B27 = 22.6% and F.B27 = 4.6%). The protein-coding variant driving the result for both amino acids was *HLA-A^∗^24:02* (significant in ALL.B27 and suggestive in M.B27, Figure 2A), with additional amino acid frequency contributed via, *HLA-A^∗^02:05* and *-A^∗^23:01* for Leu95, and *HLA-A^∗^03:02*, *-A^∗^11:01*, and *-A^∗^26:08* for Gln156. Figure 2B illustrates both amino acids relative to protein structure 3UTQ (*HLA-A^∗^02:01*). Leu95 is located on the β-sheet of the binding groove in the peptide-binding pocket F and interacts with the C-terminus of the peptide, while Gln156 is located on an α-chain which is a part of peptide binding pockets D and E (Guan et al., 2005) and interacts with three peptide positions. Due to the *HLA-A* protein-coding variant composition in our dataset, the potential amino acid combinations for each position are Ile/Val95Leu and Arg/Leu/Trp156Gln. For position 95, all options are hydrophobic, with Val having the smallest side chain. Arg156 is positively charged, while the other three amino acids available at this position are uncharged. However Leu (smallest side chain) and Trp are hydrophobic, compared to hydrophilic Gln. Both the change of charge and the size of side chain are factors that can affect peptide binding. We searched for an enrichment of the protein-coding variant containing these amino acids with respect to comorbidities, CRP levels and age at sampling, but none was significant.

## Discussion

With this study we have extended the number of MHC genes examined for a single AS population from 6 up to 15, and found nine of these significantly linked to disease. Through a nested analysis, we were also able to reveal patterns of association related to sex, and *HLA-B^∗^27* status. As we used an *n-1* genotyping methodology, the results are agnostic to individual software choice (Nordin et al., 2020). This is essential given the variability of HLA variant frequency observed when only one method is considered (e.g., *HLA-B^∗^27:05* frequency ranged between 4.8–8.0% in a study of 1000 Swedes) (Nordin et al., 2020).

The Swedish AS population examined here was largely reflective of published European datasets (Nordin et al., 2020), with the most frequent *HLA-B^∗^27* protein-coding variant being *HLA-B^∗^27:05* (97.8% cases, 7% controls). The risk conferred by this variant (ALL OR = 54.9) was one of seven significant signals we identified that had robust effect sizes in each dataset (ALL, M, and F; Figure 1). Within class I, *HLA-C^∗^01:02* and *–C^∗^02:02* added risk (ALL OR = 4.0–10.6), whilst *–C^∗^07:02* was protective (ALL OR = 0.3). These variants were in high LD with *HLA-B^∗^27* and have never been reported as independently associated. However, *HLA-C^∗^08* variants have previously been linked to *HLA-B^∗^27* positive disease (Jiao et al., 2010), but in our *HLA-B^∗^27* positive population these variants were rare (1%), and had equal frequency in cases and controls.

For class I like genes, *MICA^∗^007:01* and *MICB^∗^005:02* enhanced risk (OR = 89.5 and OR = 3.1), while *MICB^∗^004:01* was protective (OR = 0.4). The role and independence of *MICA^∗^007:01* is debated with regards to AS predisposition (Zhou et al., 2014; Cortes et al., 2018; Cortes and Brown, 2019; Zhou and Reveille, 2019). However, in our population *MICA^∗^007:01* and *HLA-B^∗^27:05* were tightly linked (ALL, *r^2^* = 0.93), and so not independently associated with disease. Interestingly, even though *HLA-B^∗^27:05* has the lowest *p*-value in our study, *MICA^∗^007:01* has the strongest effect (OR = 89.5), stronger than that noted for the 2014 discovery population (OR = 60.7) (Zhou et al., 2014). While *MICA* has been investigated in several publications (Reveille, 2014; Zhou et al., 2014; Cortes et al., 2018), *MICB* has not been equally studied, and except for suggestions of linkage via LD (Brown et al., 2002), the gene has been largely overlooked. Three *MICB* protein-coding variant conferred risk in ALL and whilst not independent of *HLA-B^∗^27*, may have a disease modifying effect. For example, the *MICB^∗^005:02* variant encodes soluble MICB, which in turn inhibits signaling through NKG2D and leads to hyporesponsive NK cells (Cox et al., 2018). It is not clear how this reduced reactiveness would increase disease risk, but the effect could be related to other immune cell interactions.

Due to genotype resolution, it was only possible to compare our results for *HLA-DRB1* and *-DQB1* with those from the large lab-type six gene MHC study (Reveille et al., 2019). In that experiment, ten class II variants were associated with disease (protective OR = 0.5–0.6 and risk OR = 1.3–2.7) (Reveille et al., 2019). We were able to replicate four variants, with similar effect sizes and same directionality, even though our population was smaller and we employed additional software programs for typing (*HLA-DRB1^∗^01:01* OR = 1.8, -*DQB1^∗^05:01* OR = 1.6, -*DRB1^∗^15:01* and -*DQB1^∗^06:02* both OR = 0.5) (Reveille et al., 2019). The inability to replicate all signals could be a reflection of study size, or population differences (Swedish versus a mixed European). For example, *HLA-DRB1^∗^04:04* had a published case/control variant frequency of 6.5/2.5% (OR = 2.72) (Reveille et al., 2019), while in our dataset it was 4.9/4.1% (OR = 1.28). Our choice of concordance methodology assisted reproducibility, but our homogenous genetic background meant some loci were largely neutral.

Our cases were predominantly male (73%), and this was likely reflected in the sharing of significant results between the ALL and M datasets (14 alleles, Figure 1). However, our smaller F set was sufficiently powered to identify three variants that increased disease susceptibility in this sex alone (*HLA-DQA1^∗^04:01, -DRB1^∗^08:01, -DQB1^∗^04:02*; Figure 1). While *HLA-DQB1^∗^04:02* and *-DQA1^∗^04:01* have not been noted previously, the presence of *DRB1^∗^08:01* has been shown to be negatively associated with AS radiographic severity (as BASRI-spine score normalized for AS duration; Ward et al., 2009). It could be that the increased frequency of this protein-coding variant in the F dataset reflects the reduced BASRI-spine score observed in female patients (Lee et al., 2007). Intriguingly, *DRB1^∗^08:01* has also been shown to enhance the effect of additional *HLA* variants in multiple sclerosis, an autoimmune disease more prevalent in women (Dyment et al., 2005). Shared genetic risk factors outside of the MHC have been reported for these diseases previously (e.g., *IL7R, PTGER4*, Cortes et al., 2013), and these results suggest additional investigations are warranted.

We used *HLA-B^∗^27* positive datasets in the attempt to identify signals independent of this key variant, and discovered novel associations between *HLA-A* and AS (*HLA-A^∗^24:02* and amino acids Ile/Val95Leu and Arg/Leu/Trp156Gln, OR = 0.3–0.4). While *HLA-A^∗^24:02* was shown to segregate with *HLA-B^∗^27:05*, the presence of nine additional *HLA-A^∗^24:02* haplotypes indicated that this protective signal was not solely driven by hitchhiking with *HLA–B^∗^27:05*. In terms of HLA-A amino acids, both Leu95 and Gln156 have the potential to interact with the peptide within the binding groove domain (Figure 2B). The Ile/Val95Leu change lies within pocket F which is critical for the binding of the peptide, and may mediate the peptide’s PΩ anchor binding ability (Bade-Doeding et al., 2007). A protective association was revealed between this amino acid and psoriasis vulgaris (Okada et al., 2014), however the mode of action was unknown. Residue 156 is part of pockets D and E, and changes here can directly impact the protein’s ability to bind peptides, and changes at Gln156 can also result in shorter than average peptide–MHC class I complexes being presented to nucleated cells (Eichmann et al., 2014). Alterations at this position have been suggested to act as part of the mechanism behind graft rejection following hematopoietic stem cell transplantation (HSCT) (Balas et al., 2017). Intriguingly, changes at both residues 95 and 156 have been shown to negatively affect the 100 days survival after HSCT, highlighting the role of these positions in the immune response (Marino et al., 2012). A recent study of HLA-DRB1 class II molecules in multiple sclerosis, suggested that *HLA* variants might act in *trans* to compensate for the effect of risk variants (Mamedov et al., 2020). In those studies, it was suggested that the protective molecule possessed the kinetic ability to discriminate between endogenous and exogenous peptide, a characteristic not present in the risk variant. This process would serve to reduce the density of functional MHC clusters and so down regulate T cell response (Mamedov et al., 2020). In the case of AS, the compensatory mechanism would be between *HLA-A^∗^24:02* and *HLA-B^∗^27*. While certain *HLA-A* and *HLA-B* variants can recognize the same epitopes (Marsh et al., 2021), it is not known if peptide kinetics could be the protective mechanism for these class I molecules. It is clear that *HLA-A^∗^24:02*, and amino acid residues 95 and 156, warrant further investigation as to their protective role in AS disease modification.

This is the largest study to search for correlations between the MHC and AS in Sweden. It is also to date, the largest MHC gene set considered for this disease. However, there are certain limitations to the current study. We used short read next generation sequencing data and the concordance results from four software programs to genotype the case and control samples considered here. As noted, our genotypes are robust, but we were unable to access a similar dataset for replication, or an imputed or lab typed AS dataset in which to replicate our findings. We also limited our investigation of AS association to the MHC protein-coding variants, and note that further clinical investigation of these results should be undertaken in the context of the nuclear genomes of the patients and controls considered.

With this work we revealed novel associations with likely clinical consequence, and confirmed the impact of several key class I and II protein-coding variants to disease. We clearly showed that clinical phenotype, sex-stratification of disease, is mirrored by the underlying genetics of AS, and suggest future studies consider the sexes separately in order to tease apart the signals that are being masked in heterogeneous populations.

## Data Availability Statement

The case and control population protein-coding variant frequency data are freely available (doi: 10.17044/scilifelab.13386653). Flat files containing per individual HLA genotyping data generated from each software program, and for the final concordance data set, are available upon request with agreement to terms and conditions for data download.

## Ethics Statement

The studies involving human participants were reviewed and approved by the Regional Committee of Linköping, Dnrs 2010/182-3 and 98110 (SweAS population), and as per cited publications (SweGen and the Uppsala Bioresource). The patients/participants provided their written informed consent to participate in this study.

## Author Contributions

JN and JM designed the project and drafted the manuscript with input from all authors. AlK, JC, PE, and PS attained and characterized the SweAS population. KT and LR performed a similar task for the Uppsala Bioresource. ÅsK, EM, AM, and JM generated sequencing data while JN, LHR, AM, and JM performed the bioinformatics analyses with statistical support from MP and insight from KL-T. All the authors revised and approved the manuscript.

## Conflict of Interest

The authors declare that the research was conducted in the absence of any commercial or financial relationships that could be construed as a potential conflict of interest.
