# Supplementary material for: Association of Protective HLA-A With HLA-B∗27 Positive Ankylosing Spondylitis
Source: Front Genet. 2021 Jul 15;12:659042. doi: 10.3389/fgene.2021.659042 (PMC8320510; doi:10.3389/fgene.2021.659042)
Supplement: Supplementary file 1 [file Data_Sheet_1.docx]

Supplementary Material

1 Supplementary Methods 1

1.1 Software methods 1

1.2 Reference 2

2 Supplementary Figures and Tables 4

2.1 Supplementary Figures 4

Supplementary Figure S1. Age distribution of cases and controls in the six test datasets. 4

Supplementary Figure S2. Overlapping comorbidity distribution in the male and female datasets. 5

Supplementary Figure S3. Pipeline used for *HLA* protein-coding allele typing and the generation of a high confidence genotyping set. 6

Supplementary Figure S4. Depth of coverage for each population (Uppsala Bioresource (UBR), grey; SweAS, blue; SweGen, red) across each gene genotyped based on transcript space. 7

Supplementary Figure S5. Intersection of variants available (A, C) and typed by each software (B, D). 8

Supplementary Figure S6. Relationship between protein-coding allele frequency called per software and the high confidence set. 9

Supplementary Figure S7. Protein-coding alleles with suggestive disease association. 10

2.2 Supplementary Tables 11

Supplementary Table S1. Genotyping summary statistics for samples, genotyping counts and variant homozygosity. 11

Supplementary Table S2. Genotyping rates across targeted and WGS data. 12

Supplementary Table S3. Summary of significant and suggestive association results across the six datasets. 13

Supplementary Table S4. Frequency of *HLA-B* segregation with haplotype *HLA-DQA1*04:01* -DQB1*04:02 -DRB1*08:01 16

Supplementary Table S5. Pair-wise linkage disequilibrium (LD) for the 15 HLA genes considered in the association tests. 17

Supplementary Table S6. Most frequent *HLA-A* – *HLA-B* haplotypes in ALL.B27 data. 18

Supplementary Table S7. Frequency of all *HLA-A*24:02* – *HLA-B** haplotypes in ALL.B27 data. 18

# Supplementary Methods

## Software methods

Four software methods were used for HLA genotyping, Supplementary Figure S3. The conditions for each are indicated below. The reference sequence for each software was sourced from the ImMunoGeneTics project/human leukocyte antigen (IMGT/HLA) database, with each software program using a different version and section from this resource (e.g. exonic or genomic).(Robinson et al., 2015) All software programs typed to at least 2-field resolution and the *n-1* method was applied at that level (Supplementary Figure S3).

**SNP2HLA** (Jia et al., 2013) is based on Beagle (Browning and Browning, 2007) and uses a Hidden Markov model and the T1DGC reference panel of 5225 Europeans(Jia et al., 2013) to impute *HLA* variants. No version for IMGT/HLA is specified, but it must be pre 3.11.0. SNP2HLA was implemented with the default settings, 10 iterations and window size of 1000 markers.

**HLA-VBSeq** (Nariai et al., 2015) remaps reads with help from a variational Bayesian approach to a user defined IMGT/HLA genomic reference, we selected v3.34.0. To gain as much information as possible, the threshold for a typed variant was relaxed (from the recommended allele coverage threshold of >20% of mean coverage to >10%). This was shown to be effective previously.(Nordin et al., 2020) Coverage for the 17 genes was calculated using the longest transcript and Picard v1.92 HS-metrics (<http://broadinstitute.github.io/picard/>). Otherwise the default settings were used.

**HLAscan** (Ka et al., 2017) uses the nucleotide sequences from IMGT/HLA v3.21.0 to realigns reads. The software utilizes a score function, ranking best match variant based on the number of unique reads mapping to each reference allele and applying a penalty for the gap size between reads. HLAscan primarily uses exon 2 and 3 for calling variants, with an extension to exon four when needed for both class I and II. Exon 5 can also be considered for class I. Variants that differed at exonic positions not used by the software, were considered as equally likely, and both options were available for the *n-1* combination. Default settings were used; score cut-off 50, constant using ScoreFunc 20.

**HLA-HD** (Kawaguchi et al., 2017) creates its own dictionary from the IMGT/HLA database and the default reference version 3.15.0 was used. The alignment of reads by Bowtie 2(Langmead and Salzberg, 2012) to the dictionary is scored based on the quality of the match and reads were weighted based on the number of mappings it does. Firstly, the software assigns G-group and then it expands to find the correct variant. Default settings were used.

## Reference

Browning, S. R., and Browning, B. L. (2007). Rapid and accurate haplotype phasing and missing-data inference for whole-genome association studies by use of localized haplotype clustering. *Am. J. Hum. Genet.* 81, 1084–97. doi:10.1086/521987.

Jia, X., Han, B., Onengut-Gumuscu, S., Chen, W.-M. M., Concannon, P. J., Rich, S. S., et al. (2013). Imputing Amino Acid Polymorphisms in Human Leukocyte Antigens. *PLoS One* 8, e64683. doi:10.1371/journal.pone.0064683.

Ka, S., Lee, S., Hong, J., Cho, Y., Sung, J., Kim, H.-N., et al. (2017). HLAscan: genotyping of the HLA region using next-generation sequencing data. *BMC Bioinformatics* 18, 258. doi:10.1186/s12859-017-1671-3.

Kawaguchi, S., Higasa, K., Shimizu, M., Yamada, R., and Matsuda, F. (2017). HLA-HD: An accurate HLA typing algorithm for next-generation sequencing data. *Hum. Mutat.* 38, 788–797. doi:10.1002/humu.23230.

Langmead, B., and Salzberg, S. L. (2012). Fast gapped-read alignment with Bowtie 2. *Nat. Methods* 9, 357–359. doi:10.1038/nmeth.1923.

Nariai, N., Kojima, K., Saito, S., Mimori, T., Sato, Y., Kawai, Y., et al. (2015). HLA-VBSeq: accurate HLA typing at full resolution from whole-genome sequencing data. *BMC Genomics* 16, S7. doi:10.1186/1471-2164-16-S2-S7.

Nordin, J., Ameur, A., Lindblad-Toh, K., Gyllensten, U., and Meadows, J. R. S. (2020). SweHLA: the high confidence HLA typing bio-resource drawn from 1000 Swedish genomes. *Eur. J. Hum. Genet.* 28. doi:10.1038/s41431-019-0559-2.

Robinson, J., Halliwell, J. A., Hayhurst, J. D., Flicek, P., Parham, P., and Marsh, S. G. E. (2015). The IPD and IMGT/HLA database: Allele variant databases. *Nucleic Acids Res.* 43, D423–D431. doi:10.1093/nar/gku1161.

# Supplementary Figures and Tables

## Supplementary Figures


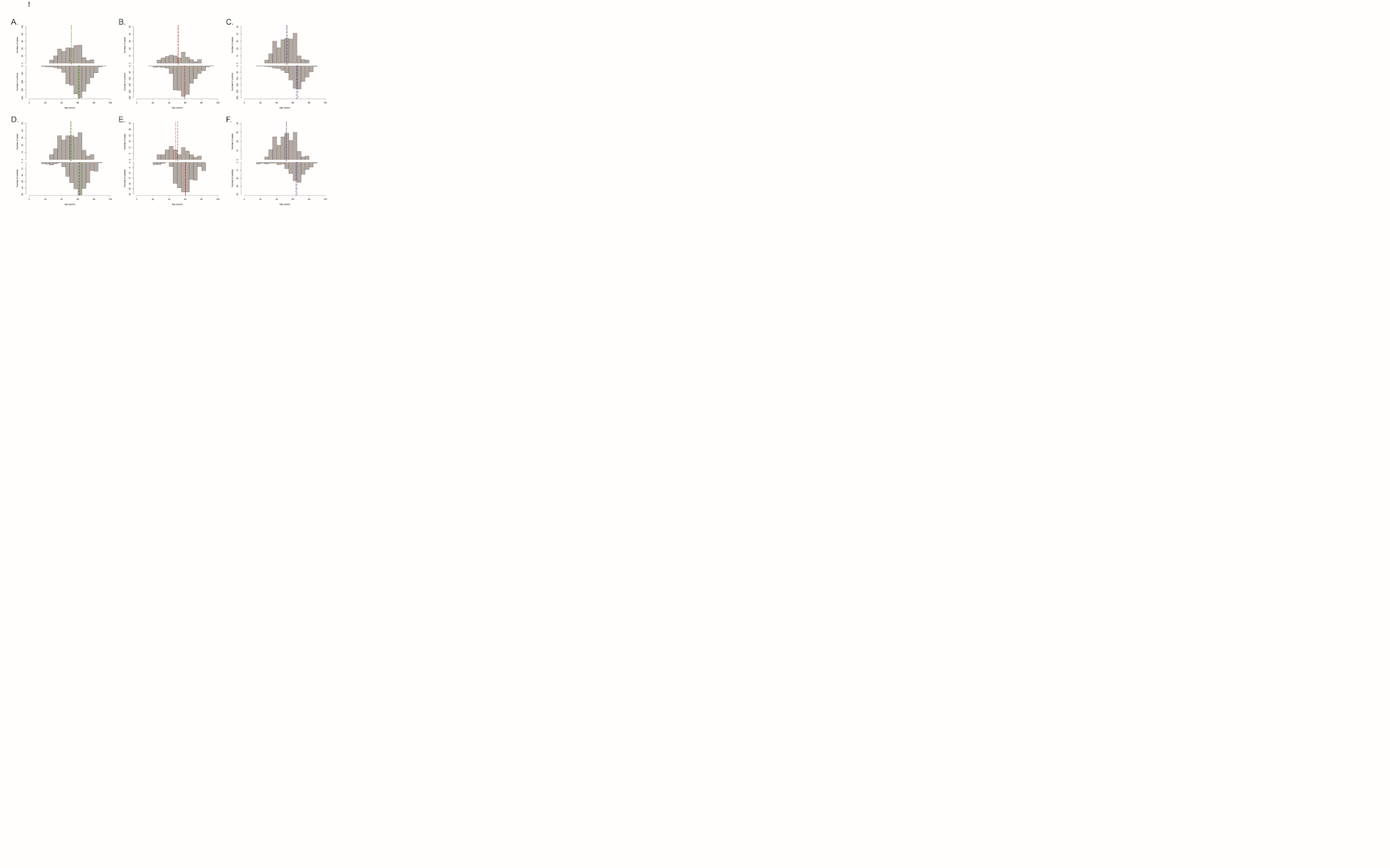


### Supplementary Figure S1. Age distribution of cases and controls in the six test datasets.

**(A)** ALL: all samples (n=2506). **(B)** F: only female samples (n=1396). **(C)** M: only male samples (n=1110). **(D)** ALL.B27: all *HLA-B*27* positive samples (n=508). **(E)** F.B27 *HLA-B*27* positive female samples (n=219) and **(F)** M.B27 *HLA-B*27* positive male samples (n=289). The lighter coloured dotted line is the median and the dark the mean value.


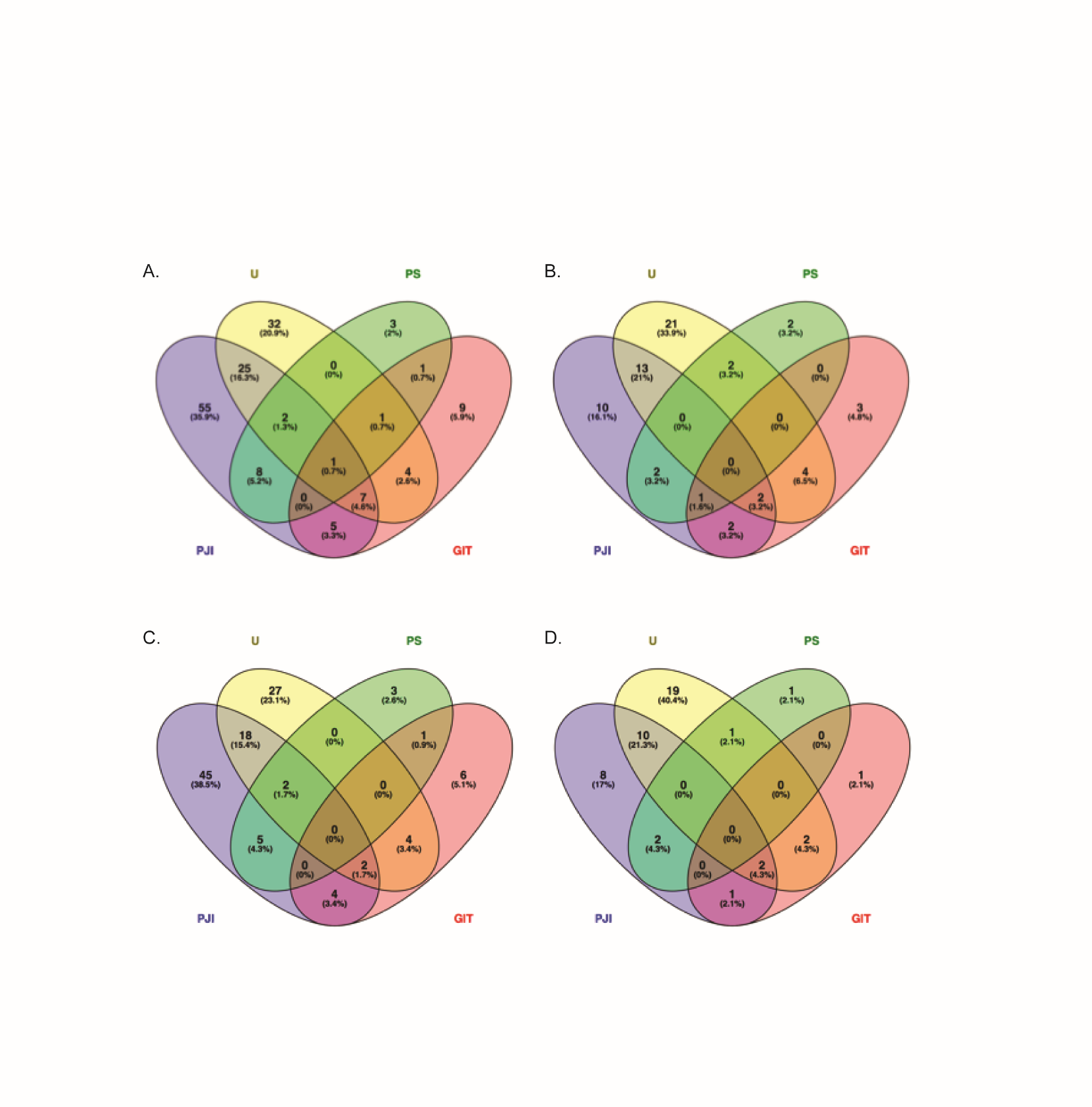


### Supplementary Figure S2. Overlapping comorbidity distribution in the male and female datasets.

**(A)** M: only male samples, **(B)** F: only female samples, **(C)** M.B27 only *HLA-B*27* positive males and **(D)** F.B27 only *HLA-B*27* positive females. U=uveitis, PS=psoriasis, PJI=peripheral joint involvement and GIT=gut involvement.

**
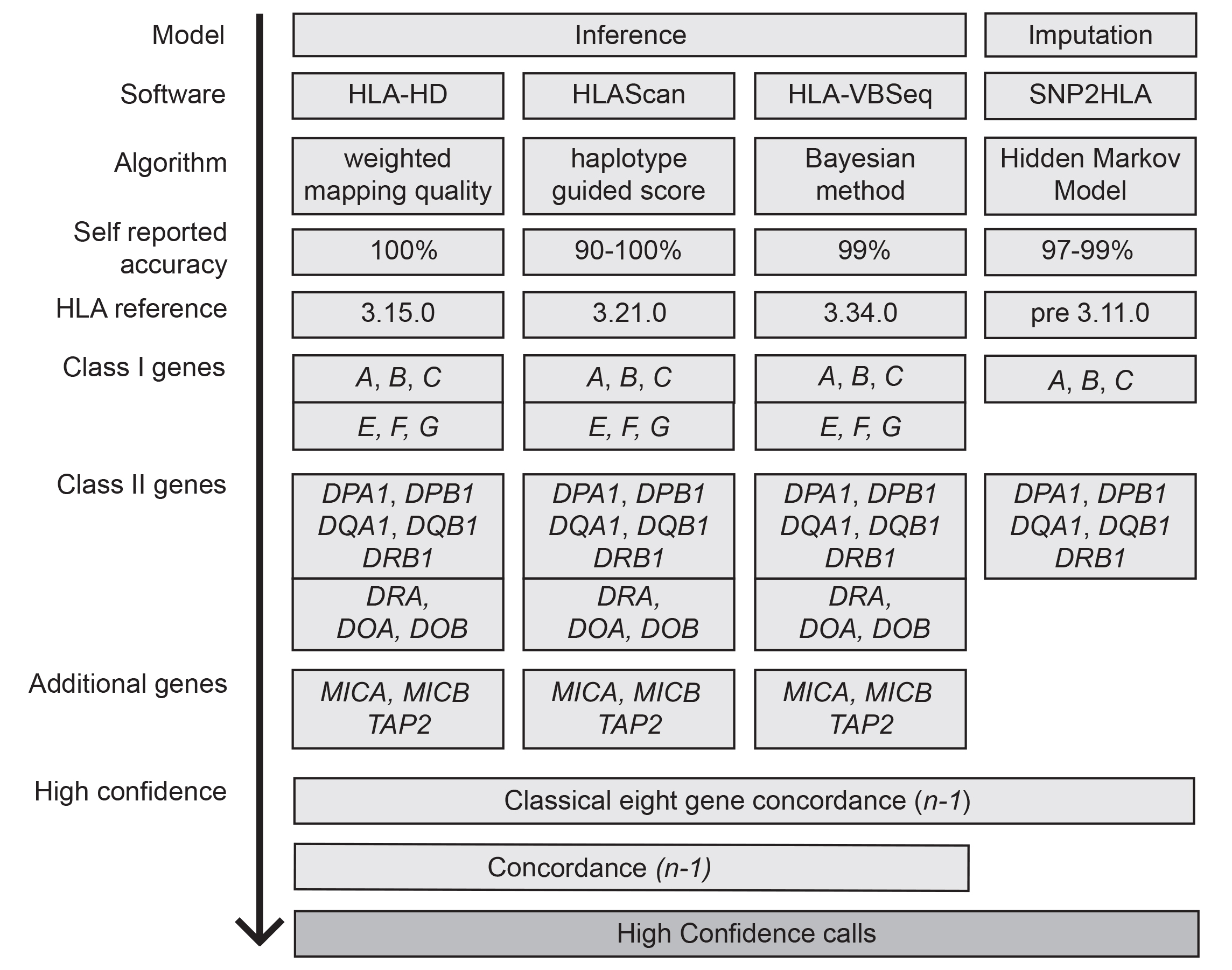
**

### Supplementary Figure S3. Pipeline used for *HLA* protein-coding allele typing and the generation of a high confidence genotyping set.

Four software programs, both inference and imputation, were used for this process. Each program’s algorithm, base *HLA* reference and number of genes available for typing, is indicated. The self reported accuracy measure for each software is also show. The High Confidence calls, were based on concordance with the *n-1* method, where the maximum number of software available for a gene was *n*.


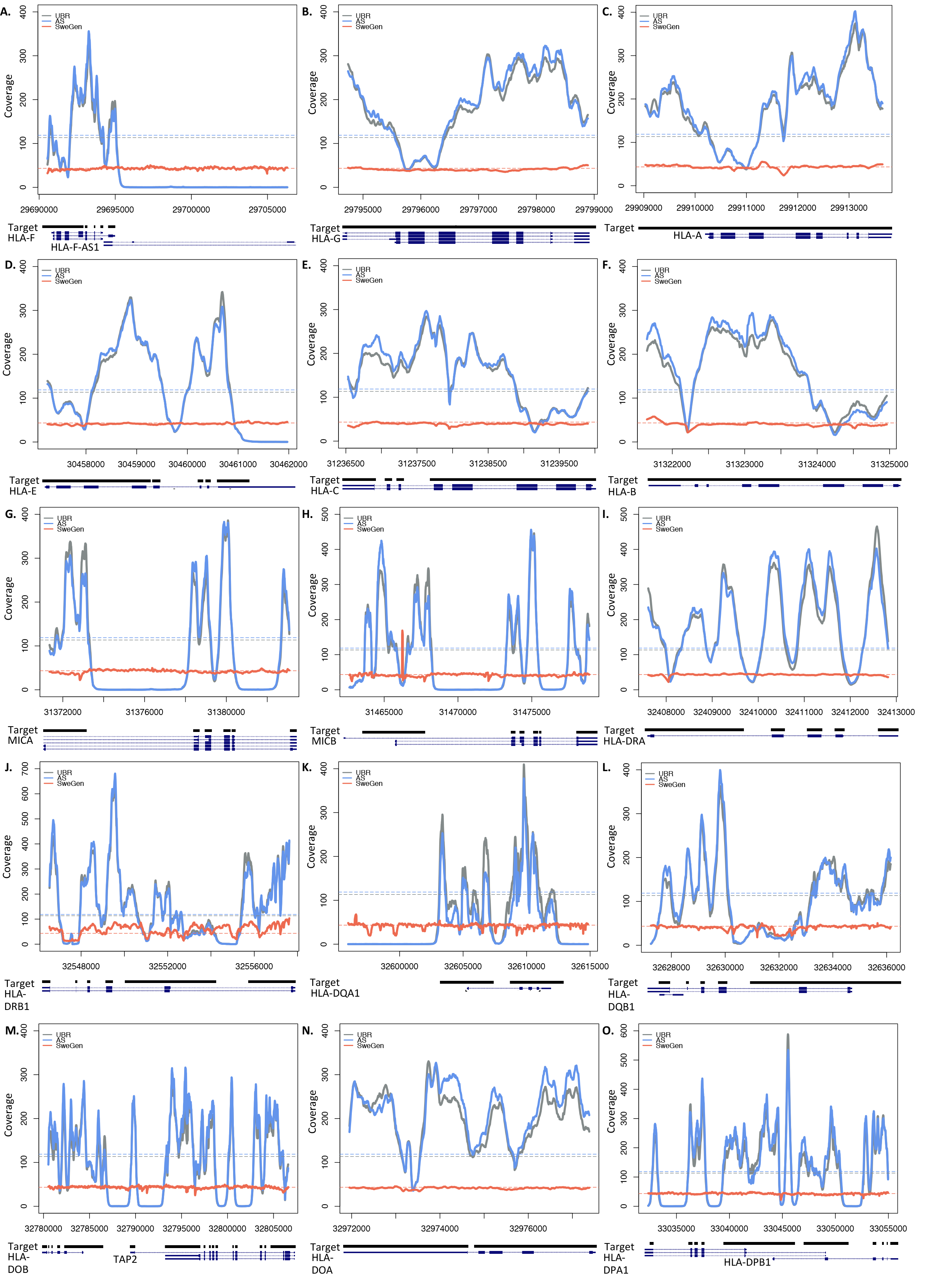


### Supplementary Figure S4. Depth of coverage for each population (Uppsala Bioresource (UBR), grey; SweAS, blue; SweGen, red) across each gene genotyped based on transcript space.

**(A)** *HLA-F*, **(B)** *HLA-G*, **(C)** *HLA-A*, **(D)** *HLA-E*, **(E)** *HLA-C*, **(F)** *HLA-B*, **(G)** *MICA*, **(H)** *MICB*, **(I)** *HLA-DRA*, **(J)** *HLA-DRB1*, **(K)** *HLA-DQA1*, **(L)** *HLA-DQB1*, **(M)** *HLA-DOB* and *TAP2*, **(N)** *HLA-DOA*, **(O)** *HLA-DPA1* and *HLA-DPB1*. The target capture space used for SweAS and UBR is shown, as are example gene RefSeq transcripts. Both the average depth across the gene (solid line, 10 bp bins) and average depth across the 17 gene space (dotted line) are indicated.

–

### Supplementary Figure S5. Intersection of variants available (A, C) and typed by each software (B, D).

*HLA-E*, *-F*, *-G*, *MICA*, *MICB* and *TAP* were genotyped with three software programs, the remaining 11 genes were typed with four (See Supplementary Figure S3).


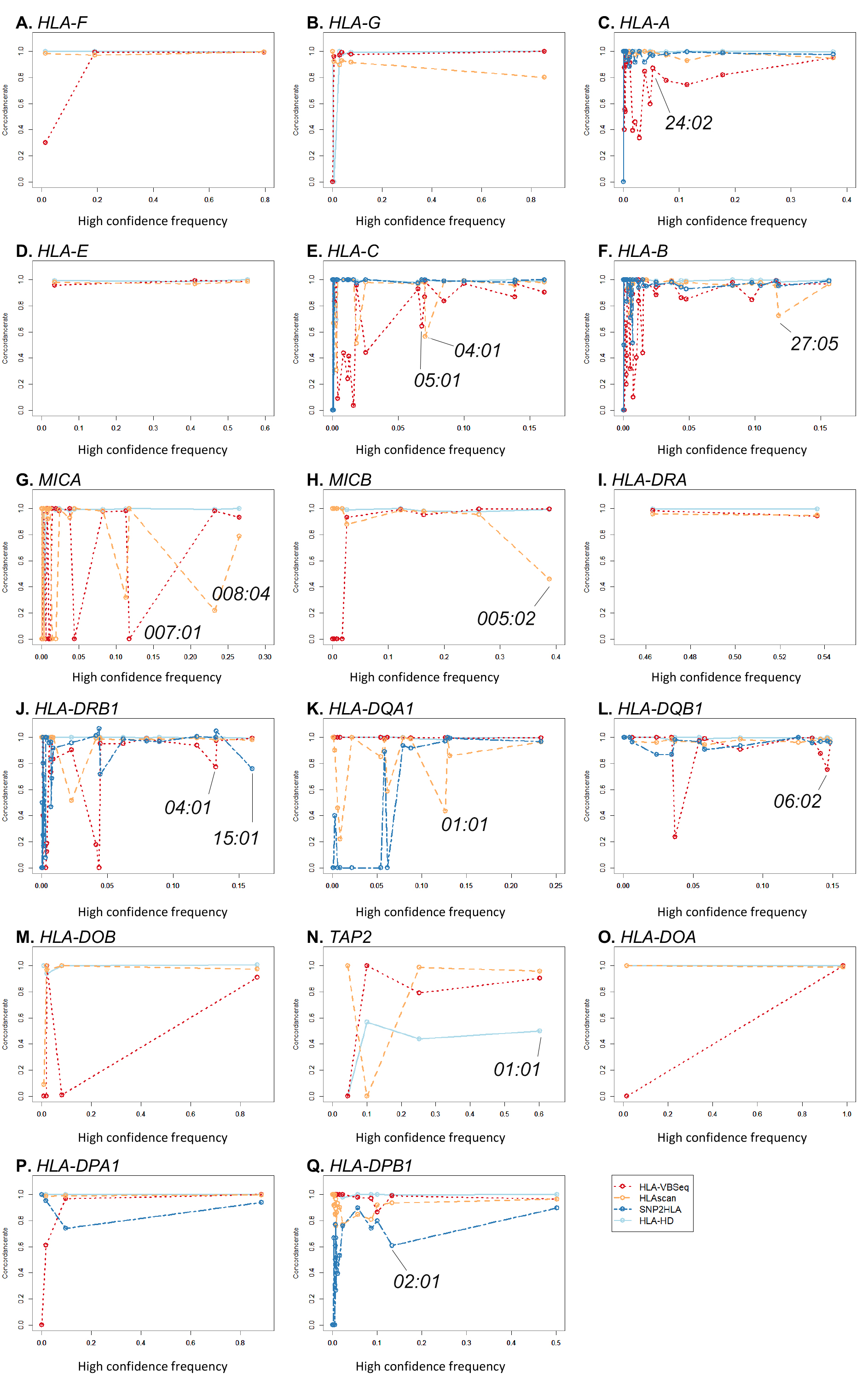


### Supplementary Figure S6. Relationship between protein-coding allele frequency called per software and the high confidence set.

**(A)** *HLA-F*, **(B)** *HLA-G*, **(C)** *HLA-A*, **(D)** *HLA-E*, **(E)** *HLA-C*, **(F)** *HLA-B*, **(G)** *MICA*, **(H)** *MICB*, **(I)** *HLA-DRA*, **(J)** *HLA-DRB1*, **(K)** *HLA-DQA1*, **(L)** *HLA-DQB1*, **(M)** *HLA-DOB*, **(N)** *TAP2*, **(O)** *HLA-DOA*, **(P)** *HLA-DPA1* and **(Q)** *HLA-DPB1*. Software used for genotyping were HLA-VBSeq (red), HLAscan (yellow), SNP2HLA (dark blue) and HLA-HD (light blue).


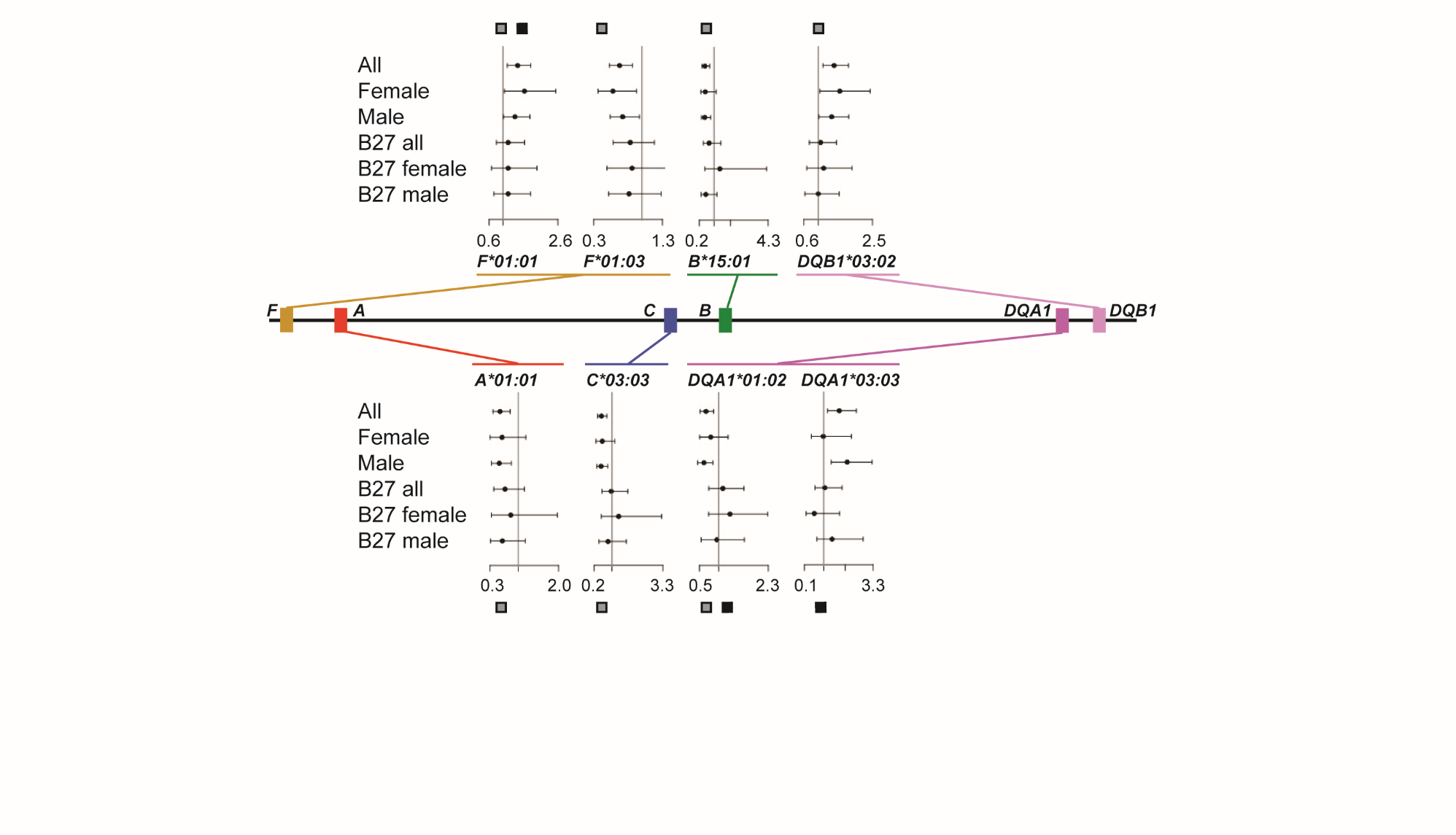


### Supplementary Figure S7. Protein-coding alleles with suggestive disease association.

The datasets with suggestive significance (adjusted p-value<0.05) is indicated with a box; All (ALL), grey; Male (M), black. No variant showed suggestive significance in the female (F) dataset. For each variant, the odds ratio (OR) and its 95% confidence interval are indicated. An OR of 1 is marked with a vertical line.

## Supplementary Tables

### Supplementary Table S1. Genotyping summary statistics for samples, genotyping counts and variant homozygosity.

|  | Samples^1^ | Genotyping Rate (%) | Variants | Homozygosity (%) |
| --- | --- | --- | --- | --- |

| Genes | All^1^ | Control | Cases | All | Control | Cases | All | Control | Cases | All | Control | Cases |
| --- | --- | --- | --- | --- | --- | --- | --- | --- | --- | --- | --- | --- |
| *A* | 2013 | 1784 | 229 | 80.3 | 81.2 | 73.9 | 29 | 29 | 18 | 16.2 | 16.3 | 15.5 |
| *B* | 2175 | 1926 | 249 | 86.8 | 87.7 | 80.3 | 40 | 40 | 26 | 7.1 | 7.8 | 2.3 |
| *C* | 2179 | 1906 | 273 | 87.0 | 86.8 | 88.1 | 25 | 25 | 16 | 10.0 | 10.2 | 5.5 |
| *DPA1* | 2381 | 2095 | 286 | 95.0 | 95.4 | 92.3 | 4 | 4 | 3 | 74.9 | 75.0 | 74.2 |
| *DPB1* | 1921 | 1714 | 207 | 76.7 | 78.1 | 66.8 | 20 | 20 | 19 | 24.1 | 24.5 | 21.3 |
| *DQA1* | 2138 | 1909 | 229 | 85.3 | 86.9 | 73.9 | 16 | 16 | 13 | 9.3 | 9.7 | 6.1 |
| *DQB1* | 2221 | 1946 | 275 | 88.6 | 88.6 | 88.7 | 15 | 15 | 14 | 10.9 | 11.0 | 10.6 |
| *DRB1* | 2071 | 1844 | 227 | 82.6 | 84.0 | 73.2 | 33 | 33 | 23 | 8.3 | 8.7 | 5.5 |
| *DOA* | 2506 | 2196 | 310 | 100.0 | 100.0 | 100.0 | 2 | 2 | 2 | 96.6 | 96.6 | 98.1 |
| *DOB* | 2326 | 2034 | 292 | 92.8 | 92.6 | 94.2 | 5 | 5 | 5 | 71.9 | 71.9 | 72.3 |
| *DRA* | 2485 | 2175 | 310 | 99.2 | 99.0 | 100.0 | 2 | 2 | 2 | 49.9 | 50.2 | 48.1 |
| *E* | 2499 | 2189 | 310 | 99.7 | 99.7 | 100.0 | 3 | 3 | 3 | 48.4 | 47.7 | 53.9 |
| *F* | 2478 | 2170 | 308 | 98.9 | 98.8 | 99.4 | 3 | 3 | 3 | 65.8 | 64.8 | 72.3 |
| *G* | 2495 | 2186 | 309 | 99.6 | 99.5 | 99.7 | 6 | 6 | 6 | 73.5 | 73.3 | 74.8 |
| *MICA* | 2293 | 2029 | 264 | 91.5 | 92.4 | 85.2 | 20 | 20 | 16 | 16.1 | 18.1 | 1.9 |
| *MICB* | 2292 | 2008 | 284 | 91.5 | 91.4 | 91.6 | 11 | 11 | 8 | 24.7 | 23.8 | 31.6 |
| *TAP2* | 1933 | 1683 | 250 | 77.1 | 76.6 | 80.6 | 4 | 4 | 4 | 30.8 | 31.0 | 29.4 |

^1^A total of 2506 samples were genotyped, 2196 controls and 310 cases. Genes indicated in red with genotyping rate < 80% were removed from downstream analyses.

### Supplementary Table S2. Genotyping rates across targeted and WGS data.

|  | Missing |  |  |  |  |  |
| --- | --- | --- | --- | --- | --- | --- |
|  | Target* |  | WGS§ |  | Combined |  |
| Gene | Count | Fraction | Count | Fraction | Count | Fraction |
| *A* | 472 | 0.313 | 21 | 0.021 | 493 | 0.197 |
| *B* | 294 | 0.195 | 37 | 0.037 | 331 | 0.132 |
| *C* | 296 | 0.197 | 31 | 0.031 | 327 | 0.130 |
| *DPA1* | 101 | 0.067 | 24 | 0.024 | 125 | 0.050 |
| *DPB1* | 451 | 0.299 | 134 | 0.134 | 585 | 0.233 |
| *DQA1* | 280 | 0.186 | 88 | 0.088 | 368 | 0.147 |
| *DQB1* | 273 | 0.181 | 12 | 0.012 | 285 | 0.114 |
| *DRB1* | 307 | 0.204 | 128 | 0.128 | 435 | 0.174 |
| *DOA* | 0 | 0.000 | 0 | 0.000 | 0 | 0.000 |
| *DOB* | 80 | 0.053 | 100 | 0.100 | 180 | 0.072 |
| *DRA* | 0 | 0.000 | 21 | 0.021 | 21 | 0.008 |
| *E* | 3 | 0.002 | 4 | 0.004 | 7 | 0.003 |
| *F* | 20 | 0.013 | 8 | 0.008 | 28 | 0.011 |
| *G* | 11 | 0.007 | 0 | 0.000 | 11 | 0.004 |
| *MICA* | 125 | 0.083 | 88 | 0.088 | 213 | 0.085 |
| *MICB* | 127 | 0.084 | 87 | 0.087 | 214 | 0.085 |
| *TAP2* | 246 | 0.163 | 327 | 0.327 | 573 | 0.229 |

*1506 samples were drawn from targeted sequencing (SweAs and UBR)

§1000 samples were drawn from whole genome sequencing (WGS) (SweGen). Genes indicated in red with genotyping rate < 80% are highlighted.

### Supplementary Table S3. Summary of significant and suggestive association results across the six datasets.

|  | ALL | | | F | | | M | | | ALL.B27 | | | F.B27 | | | M.B27 | | |
| --- | --- | --- | --- | --- | --- | --- | --- | --- | --- | --- | --- | --- | --- | --- | --- | --- | --- | --- |
| Locus | p-value | OR | 95% CI | p-value | OR | 95% CI | p-value | OR | 95% CI | p-value | OR | 95% CI | p-value | OR | 95% CI | p-value | OR | 95% CI |
| *A*01:01* | 1.80E-03 | 0.5 | 0.4-0.8 | 1.40E-01 | 0.6 | 0.3-1.2 | 5.50E-03 | 0.5 | 0.3-0.8 | 1.50E-01 | 0.7 | 0.4-1.2 | 6.50E-01 | 0.8 | 0.3-2.0 | 1.40E-01 | 0.6 | 0.3-1.2 |
| *A*02:01* | 6.40E-04 | 1.4 | 1.2-1.7 | 2.50E-02 | 1.5 | 1.1-2.2 | 9.10E-03 | 1.4 | 1.1-1.8 | 1.20E-01 | 1.3 | 0.9-1.7 | 4.40E-01 | 1.2 | 0.7-1.9 | 1.70E-01 | 1.3 | 0.9-2.0 |
| *A*24:02* | 1.10E-02 | 0.6 | 0.4-0.9 | 2.40E-01 | 0.6 | 0.3-1.4 | 2.40E-02 | 0.5 | 0.3-0.9 | 1.70E-03 | 0.4 | 0.2-0.7 | 2.50E-01 | 0.6 | 0.2-1.5 | 2.30E-03 | 0.3 | 0.2-0.7 |
| *A*31:01* | 6.30E-06 | 2.6 | 1.7-3.9 | 1.40E-03 | 3.1 | 1.5-6.2 | 8.30E-04 | 2.4 | 1.4-3.9 | 2.50E-01 | 1.4 | 0.8-2.4 | 5.20E-01 | 1.3 | 0.6-3.2 | 3.40E-01 | 1.4 | 0.7-3.0 |
| *B*07:02* | 3.80E-08 | 0.3 | 0.2-0.5 | 3.90E-03 | 0.4 | 0.2-0.7 | 2.70E-06 | 0.3 | 0.2-0.5 | 5.40E-01 | 0.8 | 0.5-1.4 | 4.10E-01 | 0.7 | 0.3-1.6 | 9.70E-01 | 1.0 | 0.5-2.1 |
| *B*15:01* | 1.10E-03 | 0.5 | 0.4-0.8 | 1.00E-01 | 0.6 | 0.3-1.1 | 4.30E-03 | 0.5 | 0.3-0.8 | 9.20E-01 | 1.0 | 0.6-1.7 | 5.70E-01 | 1.3 | 0.5-3.2 | 5.80E-01 | 0.8 | 0.4-1.7 |
| *B*27:05* | 7.10E-68 | 54.9 | 35.0-86.2 | 1.50E-25 | 26.0 | 14.1-47.9 | 4.30E-41 | 97.0 | 49.8-189.3 | 6.50E-01 | 0.8 | 0.4-1.9 | 3.30E-01 | 0.5 | 0.1-2.1 | 8.40E-01 | 1.1 | 0.4-3.2 |
| *B*44:02* | 1.10E-05 | 0.4 | 0.2-0.6 | 4.80E-02 | 0.4 | 0.2-1.0 | 7.70E-05 | 0.3 | 0.2-0.6 | 1.40E-01 | 0.6 | 0.4-1.2 | 6.90E-01 | 0.8 | 0.3-2.2 | 1.20E-01 | 0.6 | 0.3-1.2 |
| *C*01:02* | 3.30E-20 | 4.0 | 3.0-5.3 | 1.50E-10 | 5.0 | 3.0-8.1 | 1.10E-11 | 3.5 | 2.5-5.1 | 4.40E-01 | 0.9 | 0.6-1.3 | 6.60E-01 | 0.9 | 0.5-1.6 | 5.20E-01 | 0.9 | 0.5-1.4 |
| *C*02:02* | 6.30E-61 | 10.6 | 8.0-14.0 | 8.30E-19 | 8.2 | 5.1-13.0 | 1.20E-43 | 12.2 | 8.6-17.4 | 1.70E-01 | 1.3 | 0.9-1.8 | 9.00E-01 | 1.0 | 0.5-1.7 | 6.50E-02 | 1.5 | 1.0-2.4 |
| *C*03:03* | 1.20E-03 | 0.4 | 0.3-0.7 | 8.90E-02 | 0.5 | 0.2-1.1 | 5.90E-03 | 0.4 | 0.2-0.8 | 3.10E-01 | 0.7 | 0.3-1.4 | 6.10E-01 | 1.3 | 0.4-4.2 | 1.10E-01 | 0.5 | 0.2-1.2 |
| *C*04:01* | 2.70E-05 | 0.4 | 0.2-0.6 | 6.20E-02 | 0.5 | 0.2-1.0 | 1.50E-04 | 0.3 | 0.2-0.6 | 3.50E-01 | 0.7 | 0.4-1.4 | 7.60E-01 | 1.2 | 0.4-3.3 | 1.60E-01 | 0.5 | 0.2-1.3 |
| *C*05:01* | 1.00E-04 | 0.4 | 0.2-0.6 | 3.40E-01 | 0.7 | 0.3-1.5 | 9.00E-05 | 0.3 | 0.1-0.5 | 2.20E-02 | 0.4 | 0.2-0.9 | 9.90E-01 | 1.0 | 0.3-2.9 | 4.90E-03 | 0.3 | 0.1-0.7 |
| *C*07:02* | 7.00E-10 | 0.3 | 0.2-0.5 | 1.10E-03 | 0.3 | 0.2-0.6 | 1.60E-07 | 0.3 | 0.2-0.5 | 9.30E-01 | 1.0 | 0.6-1.7 | 6.20E-01 | 0.8 | 0.4-1.8 | 7.20E-01 | 1.2 | 0.5-2.5 |
| *DQA1*01:01* | 7.00E-05 | 1.7 | 1.3-2.3 | 9.70E-01 | 1.0 | 0.6-1.7 | 3.00E-06 | 2.1 | 1.6-2.9 | 9.90E-01 | 1.0 | 0.7-1.5 | 2.30E-01 | 0.6 | 0.3-1.3 | 3.90E-01 | 1.2 | 0.8-2.0 |
| *DQA1*01:02* | 2.90E-03 | 0.7 | 0.5-0.9 | 3.20E-01 | 0.8 | 0.5-1.2 | 3.40E-03 | 0.6 | 0.4-0.9 | 6.10E-01 | 1.1 | 0.7-1.7 | 3.60E-01 | 1.3 | 0.7-2.3 | 8.70E-01 | 1.0 | 0.5-1.7 |
| *DQA1*03:03* | 5.00E-03 | 1.7 | 1.2-2.5 | 9.60E-01 | 1.0 | 0.4-2.3 | 1.00E-03 | 2.1 | 1.3-3.2 | 8.70E-01 | 1.0 | 0.6-1.9 | 3.20E-01 | 0.6 | 0.2-1.7 | 3.70E-01 | 1.4 | 0.7-2.8 |
| *DQA1*04:01* | 4.00E-03 | 1.7 | 1.2-2.3 | 3.40E-05 | 3.1 | 1.8-5.3 | 4.50E-01 | 1.2 | 0.8-1.8 | 2.30E-01 | 0.7 | 0.5-1.2 | 1.40E-01 | 1.7 | 0.8-3.7 | 1.10E-02 | 0.4 | 0.2-0.8 |
| *DQB1*03:02* | 3.30E-03 | 1.4 | 1.1-1.8 | 3.00E-02 | 1.6 | 1.0-2.4 | 3.60E-02 | 1.4 | 1.0-1.8 | 7.30E-01 | 1.1 | 0.8-1.5 | 6.00E-01 | 1.1 | 0.7-1.9 | 1.0 | 1.0 | 0.6-1.6 |
| *DQB1*04:02* | 4.50E-02 | 1.4 | 1.0-2.0 | 1.30E-03 | 2.4 | 1.4-4.0 | 7.30E-01 | 1.1 | 0.7-1.6 | 7.50E-02 | 0.6 | 0.4-1.0 | 4.20E-01 | 1.4 | 0.6-2.9 | 5.50E-03 | 0.4 | 0.2-0.8 |
| *DQB1*05:01* | 1.40E-04 | 1.6 | 1.3-2.0 | 9.90E-01 | 1.0 | 0.6-1.7 | 9.00E-06 | 1.9 | 1.4-2.6 | 1.0 | 1.0 | 0.7-1.4 | 2.90E-01 | 0.7 | 0.4-1.3 | 4.40E-01 | 1.2 | 0.8-1.9 |
| *DQB1*06:02* | 1.10E-05 | 0.5 | 0.3-0.7 | 1.20E-01 | 0.7 | 0.4-1.1 | 2.10E-05 | 0.4 | 0.3-0.6 | 9.00E-01 | 1.0 | 0.6-1.6 | 9.20E-01 | 1.0 | 0.5-2.0 | 7.90E-01 | 0.9 | 0.5-1.8 |
| *DRA*01:01* | 1.30E-07 | 1.6 | 1.4-2.0 | 3.00E-01 | 1.2 | 0.9-1.6 | 1.70E-08 | 1.9 | 1.5-2.4 | 1.00E-01 | 1.3 | 1.0-1.7 | 4.10E-01 | 0.8 | 0.6-1.3 | 3.00E-03 | 1.8 | 1.2-2.6 |
| *DRA*01:02* | 1.30E-07 | 0.6 | 0.5-0.7 | 3.00E-01 | 0.8 | 0.6-1.2 | 1.70E-08 | 0.5 | 0.4-0.7 | 1.00E-01 | 0.8 | 0.6-1.0 | 4.10E-01 | 1.2 | 0.8-1.8 | 3.00E-03 | 0.6 | 0.4-0.8 |
| *DRB1*01:01* | 4.00E-05 | 1.8 | 1.4-2.4 | 2.70E-01 | 1.4 | 0.8-2.3 | 3.70E-05 | 2.0 | 1.4-2.8 | 5.90E-01 | 0.9 | 0.6-1.4 | 5.70E-01 | 0.8 | 0.4-1.6 | 8.10E-01 | 0.9 | 0.6-1.6 |
| *DRB1*08:01* | 1.60E-02 | 1.6 | 1.1-2.4 | 1.00E-03 | 2.8 | 1.5-5.3 | 3.90E-01 | 1.2 | 0.8-2.0 | 2.60E-01 | 0.7 | 0.4-1.3 | 1.90E-01 | 1.8 | 0.8-4.0 | 1.90E-02 | 0.4 | 0.2-0.9 |
| *DRB1*15:01* | 1.80E-04 | 0.5 | 0.4-0.7 | 2.00E-01 | 0.7 | 0.4-1.2 | 2.80E-04 | 0.5 | 0.3-0.7 | 8.70E-01 | 1.0 | 0.6-1.7 | 9.30E-01 | 1.0 | 0.5-2.1 | 8.80E-01 | 1.1 | 0.5-2.2 |
| *F*01:01* | 3.20E-03 | 1.4 | 1.1-1.8 | 3.10E-02 | 1.6 | 1.0-2.5 | 3.70E-02 | 1.3 | 1.0-1.8 | 4.30E-01 | 1.2 | 0.8-1.6 | 6.10E-01 | 1.2 | 0.7-2.0 | 5.40E-01 | 1.2 | 0.7-1.8 |
| *F*01:03* | 1.80E-03 | 0.7 | 0.5-0.9 | 2.10E-02 | 0.6 | 0.4-0.9 | 2.80E-02 | 0.7 | 0.5-1.0 | 3.00E-01 | 0.8 | 0.6-1.2 | 5.90E-01 | 0.9 | 0.5-1.5 | 3.70E-01 | 0.8 | 0.5-1.3 |
| *MICA*002:01* | 2.60E-04 | 0.4 | 0.3-0.7 | 1.50E-02 | 0.3 | 0.1-0.8 | 6.80E-03 | 0.5 | 0.3-0.8 | 9.70E-01 | 1.0 | 0.5-2.1 | 8.10E-01 | 0.9 | 0.3-2.9 | 8.70E-01 | 1.1 | 0.4-3.1 |
| *MICA*007:01* | 3.00E-63 | 89.5 | 52.9-151.2 | 3.70E-25 | 52.7 | 24.9-111.5 | 1.90E-38 | 128.5 | 61.7-267.7 | 9.30E-01 | 0.9 | 0.3-3.4 | 7.00E-01 | 0.6 | 0.0-7.8 | 8.90E-01 | 1.1 | 0.2-5.2 |
| *MICA*008:01* | 9.10E-07 | 0.5 | 0.4-0.7 | 2.10E-02 | 0.6 | 0.4-0.9 | 1.20E-05 | 0.5 | 0.4-0.7 | 5.10E-01 | 0.9 | 0.6-1.3 | 7.70E-01 | 0.9 | 0.5-1.7 | 5.40E-01 | 0.9 | 0.5-1.4 |
| *MICA*008:04* | 1.30E-07 | 0.5 | 0.4-0.6 | 4.70E-02 | 0.7 | 0.4-1.0 | 5.60E-07 | 0.4 | 0.3-0.6 | 3.70E-01 | 1.2 | 0.8-1.9 | 7.10E-01 | 1.1 | 0.6-2.2 | 3.90E-01 | 1.3 | 0.7-2.4 |
| *MICA*010:01* | 3.70E-04 | 0.5 | 0.4-0.7 | 8.10E-02 | 0.6 | 0.3-1.1 | 1.80E-03 | 0.5 | 0.3-0.8 | 6.20E-01 | 0.9 | 0.5-1.5 | 9.70E-01 | 1.0 | 0.4-2.7 | 5.10E-01 | 0.8 | 0.4-1.6 |
| *MICB*002:01* | 4.40E-04 | 0.6 | 0.5-0.8 | 1.30E-01 | 0.7 | 0.4-1.1 | 1.40E-03 | 0.6 | 0.4-0.8 | 8.80E-01 | 1.0 | 0.6-1.6 | 9.00E-01 | 1.0 | 0.4-2.1 | 9.20E-01 | 1.0 | 0.5-1.8 |
| *MICB*004:01* | 3.00E-13 | 0.4 | 0.3-0.5 | 2.80E-04 | 0.4 | 0.3-0.7 | 2.30E-10 | 0.3 | 0.3-0.5 | 9.30E-01 | 1.0 | 0.7-1.5 | 8.70E-01 | 0.9 | 0.5-1.8 | 9.70E-01 | 1.0 | 0.6-1.8 |
| *MICB*005:02* | 8.10E-30 | 3.1 | 2.5-3.7 | 6.70E-10 | 2.9 | 2.1-4.1 | 2.00E-21 | 3.1 | 2.5-4.0 | 4.40E-01 | 0.9 | 0.6-1.3 | 6.80E-01 | 1.1 | 0.6-2.1 | 2.10E-01 | 0.7 | 0.5-1.2 |
| Ile/Val119Leu | NA | NA | NA | NA | NA | NA | NA | NA | NA | 3.20E-04 | 0.4 | NA | 2.80E-01 | 0.6 | NA | 5.40E-04 | 0.3 | NA |
| Leu/Trp180Gln | NA | NA | NA | NA | NA | NA | NA | NA | NA | 2.10E-03 | 0.5 | NA | 6.90E-01 | 0.8 | NA | 1.80E-04 | 0.3 | NA |

OR, odds ratio. 95% CI, confidence interval

### Supplementary Table S4. Frequency of *HLA-B* segregation with haplotype *HLA-DQA1*04:01* -DQB1*04:02 -DRB1*08:01

|  | Case | | Control | |
| --- | --- | --- | --- | --- |
| Haplotype | F | M | F | M |
| *B*15:01 DRB1*08:01 DQA1*04:01 DQB1*04:02* | 0.317 | 0.246 | 0.383 | 0.371 |
| *B*18:01 DRB1*08:01 DQA1*04:01 DQB1*04:02* | 0.033 | 0.056 | 0.064 | 0.057 |
| *B*27:05 DRB1*08:01 DQA1*04:01 DQB1*04:02* | 0.383 | 0.451 | 0.078 | 0.086 |
| *B*35:01 DRB1*08:01 DQA1*04:01 DQB1*04:02* | 0.100 | 0.070 | 0.059 | 0.054 |
| *B*40:01 DRB1*08:01 DQA1*04:01 DQB1*04:02* | 0.017 | 0.007 | 0.040 | 0.051 |
| *B*40:02 DRB1*08:01 DQA1*04:01 DQB1*04:02* | 0.067 | 0.063 | 0.054 | 0.050 |
| *B*44:02 DRB1*08:01 DQA1*04:01 DQB1*04:02* | 0.033 | 0.014 | 0.069 | 0.072 |
| *B*45:01 DRB1*08:01 DQA1*04:01 DQB1*04:02* | 0.017 | 0.028 | 0.062 | 0.071 |
| *B*52:01 DRB1*08:01 DQA1*04:01 DQB1*04:02* | 0.033 | 0.007 | 0.057 | 0.055 |
| Other | 0.000 | 0.056 | 0.135 | 0.132 |

Only cases and controls with genotypes at each gene were used in the haplotype analysis, 686 females (30 cases, 683 controls), 528 males (71 cases, 528 controls).

### Supplementary Table S5. Pair-wise linkage disequilibrium (LD) for the 15 HLA genes considered in the association tests.

|  | *A* | *B* | *C* | *DPA1* | *DQA1* | *DQB1* | *DRB1* | *DOA* | *DOB* | *DRA* | *E* | *F* | *G* | *MICA* | *MICB* |
| --- | --- | --- | --- | --- | --- | --- | --- | --- | --- | --- | --- | --- | --- | --- | --- |
| *A* |  | 0.002 | 0.002 | 0.020 | 0.003 | 0.003 | 0.002 | 0.061 | 0.015 | 0.061 | 0.031 | 0.031 | 0.012 | 0.003 | 0.006 |
| *B* | NA |  | 0.000 | 0.003 | 0.000 | 0.001 | 0.000 | 0.010 | 0.002 | 0.010 | 0.005 | 0.005 | 0.002 | 0.000 | 0.001 |
| *C* | NA | NA | NA | 0.004 | 0.001 | 0.001 | 0.000 | 0.012 | 0.003 | 0.012 | 0.006 | 0.006 | 0.002 | 0.001 | 0.001 |
| *DPA1* | NA | NA | NA | NA | 0.232 | 0.232 | 0.232 | 0.697 | 0.232 | 0.697 | 0.348 | 0.348 | 0.232 | 0.232 | 0.232 |
| *DQA1* | NA | NA | NA | NA | NA | 0.001 | 0.001 | 0.018 | 0.005 | 0.018 | 0.009 | 0.009 | 0.004 | 0.001 | 0.002 |
| *DQB1* | NA | NA | NA | NA | NA | NA | 0.002 | 0.014 | 0.004 | 0.014 | 0.007 | 0.007 | 0.003 | 0.001 | 0.001 |
| *DRB1* | NA | NA | NA | NA | NA | NA | NA | 0.011 | 0.003 | 0.011 | 0.006 | 0.006 | 0.002 | 0.001 | 0.001 |
| *DOA* | NA | NA | NA | NA | NA | NA | NA | NA | 0.953 | 0.953 | 0.953 | 0.953 | 0.953 | 0.953 | 0.953 |
| *DOB* | NA | NA | NA | NA | NA | NA | NA | NA | NA | 0.660 | 0.330 | 0.330 | 0.165 | 0.165 | 0.165 |
| *DRA* | NA | NA | NA | NA | NA | NA | NA | NA | NA | NA | 0.254 | 0.254 | 0.254 | 0.254 | 0.254 |
| *E* | NA | NA | NA | NA | NA | NA | NA | NA | NA | NA | NA | 0.119 | 0.119 | 0.119 | 0.119 |
| *F* | NA | NA | NA | NA | NA | NA | NA | NA | NA | NA | NA | NA | 0.256 | 0.256 | 0.256 |
| *G* | NA | NA | NA | NA | NA | NA | NA | NA | NA | NA | NA | NA | NA | 0.124 | 0.124 |
| *MICA* | NA | NA | NA | NA | NA | NA | NA | NA | NA | NA | NA | NA | NA | NA | 0.003 |
| *MICB* | NA | NA | NA | NA | NA | NA | NA | NA | NA | NA | NA | NA | NA | NA | NA |

LD calculated for the ALL dataset using the metric *x^2^’*.

### Supplementary Table S6. Most frequent *HLA-A* – *HLA-B* haplotypes in ALL.B27 data.

| Haplotype^1^ | Case (%) | Control (%) |
| --- | --- | --- |
| *A01:01 - B08:01* | 5.7 | 5.7 |
| *A01:01 - B27:05* | 6.0 | 4.5 |
| *A02:01 - B27:05* | 19.1 | 20.8 |
| *A02:01 - B44:02* | 3.6 | 5.4 |
| *A03:01 - B27:05* | 7.9 | 7.5 |
| *A24:02 - B27:05* | 3.0 | 8.1 |
| *A31:01 - B27:05* | 8.7 | 6.1 |
| Other | 45.9 | 41.9 |

^1^Haplotypes observed at ≥ 5% in 183 cases or 442 controls are reported. All other haplotypes are condensed into “Other”.

### Supplementary Table S7. Frequency of all *HLA-A*24:02* – *HLA-B** haplotypes in ALL.B27 data.

| Haplotype^1^ | Case (%) | Control (%) |
| --- | --- | --- |
| *A24:02 - B07:02* | 0.0 | 1.1 |
| *A24:02 - B18:01* | 0.3 | 0.2 |
| *A24:02 - B27:05* | 3.0 | 8.1 |
| *A24:02 - B35:01* | 0.0 | 0.5 |
| *A24:02 - B37:01* | 0.0 | 0.2 |
| *A24:02 - B39:01* | 0.3 | 0.0 |
| *A24:02 - B40:01* | 1.1 | 0.9 |
| *A24:02 - B44:02* | 0.0 | 0.2 |
| *A24:02 - B51:01* | 0.3 | 0.0 |
| *A24:02 - B55:01* | 0.3 | 0.2 |

^1^Haplotypes frequencies calculated using 183 cases and 442 controls.
